# MicroRNAs as Targets for Cancer Diagnosis: Interests and Limitations

**DOI:** 10.34172/apb.2023.047

**Published:** 2022-07-02

**Authors:** Behrouz Shademan, Vahidreza Karamad, Alireza Nourazarian, Sepideh Masjedi, Alireza Isazadeh, Fatma Sogutlu, Cigir Biray Avcı

**Affiliations:** ^1^Department of Medical Biology, Faculty of Medicine, EGE University, Izmir, Turkey.; ^2^Department of Basic Medical Sciences, Khoy University of Medical Sciences, Khoy, Iran.; ^3^Department of Cellular and Molecular Biology Sciences, Tonekabon Branch, Islamic Azad University, Tonekabon, Iran.; ^4^Immunology Research Center, Tabriz University of Medical Sciences, Tabriz, Iran.

**Keywords:** MicroRNA, Biogenesis of miRNA, Cancer diagnosis, Cancer prognosis

## Abstract

MicroRNAs are small RNAs with ability to attach to the large number of RNA that regulate gene expression on post-transcriptional level via inhibition or degradation of specific mRNAs. MiRNAs in cells are the primary regulators of functions such as cell growth, differentiation, and apoptosis and considerably influence cell function. The expression levels of microRNAs change in human diseases, including cancer. These changes highlight their essential role in cancer pathogenesis. Ubiquitous irregular expression profiles of miRNAs have been detected in various human cancers using genome-wide identification techniques, which are emerging as novel diagnostic and prognostic cancer biomarkers of high specificity and sensitivity. The measurable miRNAs with enhanced stability in blood, tissues, and other body fluids provide a comprehensive source of miRNA-dependent biomarkers for human cancers. The leading role of miRNAs as potential biomarkers in human cancers is discussed in this article. In addition, the interests and difficulties of miRNAs as biomarkers have been explored.

## Introduction

 Cancer is a group of diseases that involve abnormal cell growth with the possibility of invasion or spread to other parts of the body. Genetic, epigenetic, and environmental factors may play a role.^1^ There are many changes in the tissue and cell surface of the tumor, so the exact mechanism of the pathogen is unknown.^2^ The complexity of cancer has increased with the discovery of different classes of genes involved, especially tumor inhibitor genes and molecular signaling pathways.^3^ Cancer is the second leading cause of death after heart disease.^4^ Of the causes of death from cancer, it is detected at an advanced stage in more than 50% of cases. A strong correlation has been found between the time of diagnosis and the life expectancy of patients. Therefore, an early cancer diagnosis is the best way to reduce the mortality rate and control it long-term.^5,6^

 MicroRNAs (miRNAs) are small non-coding RNAs (ncRNAs) that control gene expression at the post transcription level by targeting messenger RNAs (mRNAs).^7^ The new types of “oncomiRs” or “tumor suppressors” are called irregular miRNAs, which play a significant role in cancer development and progression.^8^ Studies show that more than half of the proteins encoding proteins are controlled by miRNAs. One miRNA can affect multiple genes, and multiple miRNAs can regulate one gene. Thus, a particular miRNA can control various cellular processes such as apoptosis, growth, and cell proliferation.^9-11^ The impact of microRNAs on human cancers was first identified in chronic lymphocytic leukemia studies.^12^ This evidence is growing and has led to the introduction of miRNAs as very attractive new biomarkers for many human diseases, including cancer. Irregular expression profiles of miRNAs have been detected in various human cancers, indicating the considerable possibility of brand-new biomarkers and cancer diagnosis with high sensitivity and specificity.^13^ The expression profile of miRNAs has been shown to differ between normal, and tumor tissues and these changes can be used to distinguish tumor tissues from normal tissues.^14^ the early detection of neoplasms can ensure the selection of the most effective, modern therapy, extend the time to disease progression, extend the overall survival time, and improve its quality, and early detection may be possible thanks to miRNA molecules. In this study, recent advances in cancer diagnosis and abnormalities in tumor-associated miRNAs as diagnostic biomarkers for cancer were reviewed. The advantages and disadvantages of using miRNA biomarkers as a tool for cancer diagnosis were then discussed.

## Current diagnosis strategies for cancer

 Cancer is a genetic disease that results from uncontrolled cell division. It can be caused by environmental factors and genetic abnormalities.^15^ Several critical genes, including oncogenes and tumor-inhibitory genes, play a role in cells becoming cancerous.^16^ When the disease is diagnosed at an early stage, treatment options are more effective. Cancer cells can be detected in several ways. Methods of cancer diagnosis include imaging, laboratory tests, and tumor biopsy.^17^ There are several imaging techniques that a physician can use to examine and detect cancerous tissue. Most breast cancers originate in the mammary gland tissue, which is associated with anatomical changes in the duct and the appearance of small and large masses.^18^

 One of the most effective ways to combat this disease is to diagnose it in its early stages. Studies have shown that more effective treatment can reduce the potential mortality rate if the cancer is diagnosed early.^19^ Although mammography is currently one of the most effective screening tools for breast cancer, it does not detect all breast cancers.^20^ Mammography is very difficult to interpret because the natural tissue of the breast is different and unique in each patient.^21^ Although mammography is the standard method of diagnosing breast cancer, complementary ultrasonography (complementary screening) can increase the sensitivity of the diagnosis, especially in the younger age group.^22^ Ultrasound is a painless, non-invasive procedure to examine internal organs. This device records the image of the internal organs with sound waves, which reveals the condition of the internal organs.^23^ Therefore, it is helpful to find malignant tumors hidden in the abdominal and pelvic cavities, such as gastric tumors and ovarian cancer. However, cancer diagnosis by this method is relative.^24^ Computerized tomography (CT) is used to find tumors in the body cavities that are not detected by regular clinical examinations for early detection of lung, head, and neck cancers.^25^ The amount of radiation used in this method has side effects.^25^ Magnetic resonance imaging (MRI), which uses radio waves instead of X-rays, images soft (non-bony) tissue. However, MRI is not a routine test for the definitive diagnosis of cancer.^26^ Samples for cancer diagnosis are taken through a simple outpatient procedure, usually under local anesthesia. Sometimes the sample is taken with a needle from the deep parts of the mass.^27^ However, the anesthesia, the surgery, the removal of the mass, and the associated removal of large amounts of healthy tissue have clear and unclear consequences for the body. Some believe that the formation of a cancerous mass in the body is a defensive measure by the body to control and limit cancer cells. Removal and elimination of this mass may have unintended consequences on the spread of cancer in the body.^28^

 With the advent of Next Generation Sequence (NGS) technology, gene sequencing and cancer diagnosis have entered a new field in the last decade. NGS technology can be used to diagnose many neurological diseases and cancers.^29,30^ Cancer is a very heterogeneous disease, and different cells are found in a tumor. The primary mutation that causes malignancy may only be found in a small portion of the cell genome that NGS can detect, although detection requires great care and concentration.^31^ However, since many genetic alterations are not detected in various cancers, the use of NGS is limited. Since accurate gene profiles for various cancers are not available,^31,32^ miRNAs play an essential role as biological biomarkers in cancer and can be considered for cancer screening and early detection. However, several studies are required to profile miRNAs in different types of cancer.

## MicroRNAs

 MicroRNAs (miRNAs) are a group of gene expression regulators that are synthesized endogenously and can affect the expression of genes. These molecules are single-stranded and non-coding RNAs, so the length of these miRNAs does not exceed about 23 nucleotides in the functional state.^33^ The critical point is that one miRNA can target multiple mRNAs, thus exerting a regulatory effect on different genes. Moreover, studies have shown that one mRNA is regulated by multiple miRNAs.^34^ miRNA expression is different in different tissues. This difference in expression is observed in incredibly healthy and cancer cells, indicating the importance of miRNAs in the pathogenesis process.^35^ Some miRNAs act as tumor suppressors or oncogenes by targeting cancer-related genes. They also play essential roles in apoptosis, proliferation, migration, and cell invasion.^36,37^ MiRNAs decrease target gene expression by decreasing the stability of mRNA and preventing its translation.^38^ By decreasing the abundance of specific proteins, miRNAs exert control effects on many physiological processes. Not surprisingly, their dysregulated expression can influence neoplastic diseases.^39^

## Biogenesis and function of miRNA

**Figure 1:**
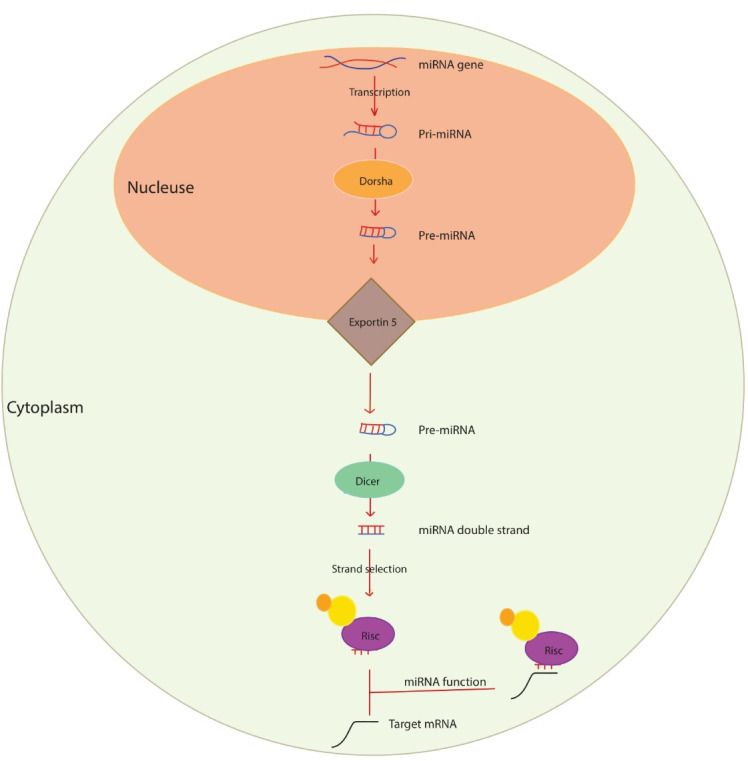
The biogenesis and function of miRNA. miRNA biogenesis takes place in the nucleus; Pri-miRNA is converted by RNase III endonuclease, Drosha, and its Cofactor (Dgcr8) into smaller stem-looped structures called precursor miRNA (pre-miRNA). Pre-miRNAs are transported by the exportin 5 from the nucleus to the cytosol; further processing results in the generation of mature miRNA by a second RNase III enzyme and Dicer

 For the first time, a miRNA gene named lin-4 was identified in Caenorhabditis elegans, which binds to the mRNA of the lin-4 construct and inhibits its translation.^40^ Later, another miRNA named let-7 was identified in Caenorhabditis Elegans, which regulates cell growth and development.^41^ MiRNAs are processed and produced in several steps: In the first stage, RNA polymerase II transcribes miRNA genes (in most cases) or RNA polymerase III, which is the product of an extended transcript (hundreds to several thousand nucleotides) called primary miRNA (pri-miRNA).^33^ Pri-miRNA is seen as a stem-loop hairpin structure with a cap ‘5 and a poly-A tail.^42^ Subsequently, a ribonuclease enzyme called Drosha cleaves the pri-miRNA with the help of DGCR8. A pre-miRNA molecule of about 60-100 nucleotides is formed with a stem-loop or hairpin structure.^42,43^ Ran GTP and exportin-5 transfer pre-miRNAs from the nucleus to the cytoplasm. Upon entering the cytoplasm, RanGTP is hydroxylated to RanGDP, leading to the release of pre-miRNA from exportin 5.^44^ The ribonuclease III, called Dicer, binds with another protein to the pre-miRNA and generates the mature miRNA by cleavage in the cytoplasm. Mature miRNA (miRNA/miRNA*) with about 22 nucleotides is a double-stranded molecule without a stem-loop hairpin structure.^45,46^ Not all bases are paired in miRNA / miRNA*, and incomplete connections between two strands can be seen. In the next step, one of the duplex miRNA strands, miRNA/miRNA*, is included in the RISC set, and the other miRNA* strand is separated or destroyed. A strand with a “5” end paired with another strand with lower stability enters the RISC complex.^9,47^ The primary role of the RISC complex is to guide the mature miRNA to the target transcript and prevent the translation process and protein production (Figure 1).

 The mature miRNA is associated with the miRNA-induced silencing complex (miRISC), which leads to post-transcriptional gene silencing. Overall, the function of miRNAs is to silence genes.^48^ There is at least one miRNA binding site in 30% to 80% of coding protein genes, so a miRNA can regulate the expression of numerous mRNAs by partially binding to an mRNA.^49,50^ MiRNA is also involved in almost all pathological and biological processes.^51^ MiRNAs specifically identify mRNA and regulate gene expression through several post-transcriptional mechanisms: (1) inhibition of translation and also (2) degradation of mRNA.^52^ The component of the regulatory mechanism depends primarily on the degree of miRNA-mRNA complementarity. When the degree of complementarity between miRNA and mRNA is high, damage to the target mRNA is possible. However, if the complementarity between miRNA and mRNA is low, the mechanism of translational repression is triggered.^53^ For this reason, changes in mi expression are observed in various diseases.

## The role of miRNAs in cancer

 MiRNAs have physiological contributions to various vital processes, both biological and pathological, and to cellular stress. Estimates suggest that conserved miRNA target regions comprise the most damaged DNA checkpoint and repair genes.^54^ High transcription rates and low translation rates can occur in genes that are primarily regulatory or important for cell functionality.^55^ Negatively modulated miRNAs allows the cell to produce many mRNA transcripts with a small amount of protein with thorough regulation.^56^ The miRNA expression profile suggests that the expression of many miRNAs is drastically altered in human cancers; undoubtedly, primary overexpression or loss occurs for some miRNAs in tumors compared to intact tissues. Tumor growth is promoted by oncogenic miRNAs, known as oncomiRs, by negatively affecting tumor-suppressing genes, and tumor-suppressing miRNAs, known as anti-oncomiRs, target oncogenes to inhibit tumor growth.^57^ MiRNAs related to the DNA damage response (DDR) pathway contribute to tumorigenesis and development. Genes related to cell cycle regulation have been shown to repress three members of the miR-34 family - miR-34a, miR-34c and miR-34b.^58^ It has been reported that miR-17/20 and miR-221/ 222 clusters target cell cycle regulators, leading to the regulation of cell cycle checkpoints and progression.^59,60^ Currently, there is evidence for functional enhancement of several miRNA target genes associated with DDR pathway. In cancer, aberrantly expressed miRNAs are detected by mature or precursor miRNA copies that are abnormally expressed compared to the corresponding intact tissues. There is increasing evidence for a typical aberrantly described profile of miRNAs alongside pre-miRNAs in human cancers. Various high-performance sequencing platforms for whole-genome analysis of miRNA gene expression have in recent years uncovered aberrantly expressed profiles of miRNAs that are either down- or up-regulated in a variety of selected human malignancies, including breast,^61^ colorectal,^62^ and prostate cancers,^63^ as well as glioma (Table 1).^64^

**Table 1:** MicroRNAs are involved in the cancer-causing process

**Type of cancer**	**microRNA**	**Target gene**	**Role in cancer**	**Reference**
Liver cancer	miR-503	VEGF-A and FGF2	Inhibition of angiogenesis	^65^
Ovarian cancer	miR-125b	HIF-1a, VEGF, HER2, HER3	Inhibition of angiogenesis	^66^
Bladder cancer	miR-200 family (miR-200a/200b/200c/141/429)	ZEB	EMT/MET process	^67^
Colorectal cancer	miR-34a	Snail, ZNF281, IL-6R	EMT process	^68^
Breast cancer	miR-335	SOX4, TNC	migration and invasion	^69^
Gastric cancer	MiR-503	Notch and IGF1R	EMT process	^70^
Non-small cell lung cancer	MiR-194	BMI-1	migration and invasion	^70^
Glioma cancer	MiR-128	E2F3a	cell growth	^71^

EMT, Epithelial-mesenchymal transition; MET, mesenchymal-epithelial transition.

## MiRNAs and their extracellular stability

 The suitability of circulating miRNAs as biomarkers, particularly for cancer, depends on their stability and ability to indicate tumor status and anticipate therapeutic responses. Several systematic studies have shown that circulating miRNAs maintain their stability even when exposed to harsh conditions that typically weaken RNAs, such as high or low pH, boiling, and prolonged storage followed by freezing and thawing cycles.^72^ The remarkably stable miRNAs have been attributed in part to their relationship with protein complexes, and these miRNAs in circulating microvesicles are referred to as exosomes. In the present study, most circulating miRNAs in plasma were shown to be co-fractionated with Argonaute2 (Ago2), suggesting that the mechanisms of action involved in the stability of miRNAs in plasma are circulating Ago2 complexes.^73^ Ago2 is a member of an RNA-mediated silencing complex. According to the findings of this recent research, the crucial effector protein of miRNA-induced silencing suggests that the vesicle-related and Ago2 complex-related miRNAs originate in different cell types and show the expression of miRNAs depending on the cell shape.

 Nevertheless, circulating vesicle-bound miRNAs accounted for only 10% of plasma. Furthermore, the entire detection was confirmed by ultrafiltration of extracellular miRNAs with Ago2. After ultracentrifugation at 110 000 g, most miRNAs in plasma and cell culture remain in the supernatant, suggesting that extracellular miRNAs emerge as non-vesicular ones.^74^ Moreover, non-miRNA species (U6 RNA, RNU24, RNU43, RNU44, RNU48, and RNU6B) and mRNAs have no connection with Ago proteins present in the extracellular milieu, present only at low levels. Some other proteins in the supernatant after Ago2 immunoprecipitation suggest a possible link with circulating miRNAs.^73^ The discovery of another mechanism, likely involving the nSMase2 pathway, shows that high-density lipoproteins can transfer circulating miRNAs and alter gene expression by transporting miRNAs to recipient cells.^75^

 However, miRNAs could additionally be associated with paracrine and autocrine miRNA signaling with exosomes. Existing evidence using only samples from intact pens or culture media suggests that most circulating miRNAs lack exosomes.^73,76^ Studies did not examine two circulating miRNA populations (i.e., extracellular and exosomal) or compare intact pens with cancer patients. Notable results of such studies show that tumor-derived exosome levels are increased in plasma samples from cancer patients compared to those in healthy donor samples.^77,78^ In addition, miRNA-containing exosomes were present in the bloodstream and various other body fluids, such as saliva.^79,80^ A research report suggests that exosomal miRNAs, which remain hidden in the bone marrow stroma, are associated with breast cancer cells and breast cancer recurrence and poor prognosis. In the current study, the contribution of miRNAs to breast cancer cell quiescence was demonstrated by visualizing their transition through intercellular interaction at gap junctions and stroma-derived exosomes between bone marrow stroma and quiescent breast cancer cells.^81^ The origin of extracellular circulating miRNAs is in dead cells, as Ago2/miRNA complexes with incredible stability are present in the cell cytoplasm.^74,82^ There is no doubt that miRNAs are produced in body fluids by tumor cells undergoing apoptosis and necrosis and by other sources, including blood cells, the liver, lungs, kidneys, and other body organs with which blood plasma remains in high contact.^83^ Theoretically, this suggests the need for warning when extracellular circulating miRNAs are used as biomarkers. This results from the fact that cancer-specific miRNAs can be hidden in circulating miRNAs from intact tissues. Studies are needed to determine the true origin, mode of action of formation in circulation, and biological effects of these miRNA molecules in distant regions.

## MiRNAs as biomarkers of diagnosis for cancer

 As previous observations have shown, cancers of various hematopoietic and epithelial lineages possess complete miRNA profiles that differ in their indications based on their developmental basis. For example, a microarray platform was used to assess the expression levels of 47 miRNAs in 101 formalin-fixed and paraffin-implanted samples from primary or metastatic cancers. Overall accuracies of 100 and 78% were obtained for primary and metastatic cancers, respectively. By applying the signature to a dataset of 170 samples with independent publications, 86% of correctly predicted metastatic cases were confirmed.^84^ Accurate determination of tumor subtypes in patients with malignancies has a significant impact on treatment decision-making. For example, a group of researchers developed a category system for renal cell carcinoma (RCC) with decision trees capable of distinguishing different RCC subtypes in 94 different subtype samples by analyzing exclusive miRNA signatures.^85^ The system was 97% sensitive at discriminating between healthy samples and RCC patients. For four RCC types (clear cell RCC (ccRCC), papillary RCC (pRCC), oncocytoma, and chromophobe RCC (chRCC)), 100% accuracy was achieved for the ccRCC subtype against a variety of other subtypes, 97% for the pRCC subtype against oncocytoma and chRCC, and 100% accuracy for distinguishing oncocytoma from chRCC.

**Figure 2:**
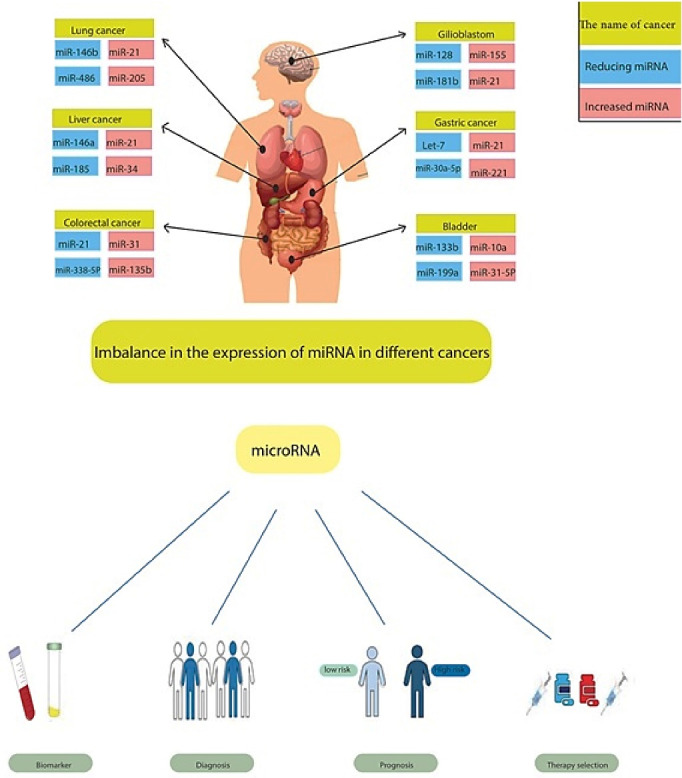
The effect of microRNAs on the clinical treatment of cancer. Since microRNA expression patterns are altered in cancer compared to normal tissues and vary between subtypes, they are likely future biomarkers for stratification and diagnosis prediction in cancer patients. In future cancer treatment, microRNAs could play an essential role in deciding which drugs to select for a patient and whether the patient will respond to the drug

 In addition, Gilad et al confirmed a miRNA-based assay to distinguish between four primary forms of lung cancer cells in an independent composite of 451 samples. Their results showed an overall accuracy of 94% for over 90% of the samples, and pathological and cytological samples showed the same performance.^86^ The observations suggest that miRNA signatures accurately categorize different types of cancer in cytological and pathological samples. Biomarkers are urgently needed for the timely diagnosis of cancer, as human survivability and diagnosis depend entirely on clumping at the time of detection. Diagnosis is mainly improved by timely diagnosis. Reports suggest that miRNA signatures are a promising option for timely diagnosis. A report on the primacy of overexpressed miR-21 and miR-205 in ductal adenocarcinomas over phenotypic regulation in ducts shows that aberrantly produced miRNAs are an initial manifestation of cancer progression,^87^ which also supports their potential application for early detection of cancer (Figure 2).

## MiRNAs as cancer prognosis biomarkers

 In addition to original tissue differentiation, subtypes, and timely cancer diagnosis contributing to cancer detection, miRNA signatures are of additional value for cancer prognostication.^88^ A signature of five miRNAs in lung cancer has also been established, which can be used to predict therapeutic consequences in non-small cell lung cancer. Highly expressed let-7a leads to many diagnoses, while elevated miR-137, miR-372 and miR-182 are associated with poor prognosis.^89,90^ In addition, plasma concentrations of miR-10b and miR-373 were investigated in a recent study on the role of metastatic breast cancer. The relationships between these miRNAs were revealed in detecting lymph node metastasis, highlighting the potential of prognostic biomarkers.^91^ Even a single miRNA can have accurate predictive potential, as studies in breast cancer patients have shown. It was found that overexpressed miR-210 was associated with an increased risk of recurrence and a lower chance of survival without recurrence. A single miR-210 level could predict diagnosis to a similar extent as a 76-gene mRNA signature assay (GENE76).^92,93^ There was a negative association between miR-410 signature and overall survival in progressive serous ovarian cancer.^94^ Incredibly, a recently discovered eight-miRNA signature model in gastric cancer can predict both overall survival and recurrence-free survival.^95^ A six-miRNA-based classifier found in patients with stage II colon cancer was an independent predictor of disease recurrence (Figure 2).^96^

## Limits of miRNAs Biomarkers of cancer diagnosis and prognosis

 MiRNAs present in the blood circulation can be quantified from different sources of substances (i.e., plasma, serum, and whole blood),^97^ demonstrating the influence of blood cell miRNAs (present in erythrocytes, leukocytes, and platelets) on the analysis of circulating miRNAs. According to their data, blood cells contribute significantly to miRNAs in the circulation and, in particular, alter specific miRNA levels. Therefore, it is impossible to consider the whole blood as a specific biological fluid to detect circulating miRNAs. Nevertheless, it is necessary to eliminate cellular components that may interfere with accurate miRNA measurement when assays are performed on plasma and serum. Several research teams have compared different plasma/serum preparation techniques, evaluating the differences between the two biological fluids in the distribution of miRNA levels.

 However, indisputable evidence is not available.^98,99^ EDTA and citrate are anticoagulant chemicals commonly added to blood collection tubes that can detect miRNAs adequately without further treatments.^100^ Nevertheless, EDTA tubes are preferable to citrate use, as the latter can lead to hemolysis (see the section on hemolysis affecting the analysis of miRNAs).^101^ Finally, a comparison of fresh and iced liquids has yielded stunning results.

 Since miRNAs are very stable in the bloodstream, no or only minor differences were observed between fresh and frozen samples, even after repeated cycles of freezing/thawing.^102,103^ However, it is recommended to avoid excessive freezing/thawing. In the case of the occurrence of degraded miRNAs (even to a limited extent), it is possible to miss miRNAs with misrepresentation. After obtaining the data, normalization is the next challenge. Techniques can be expected to vary between samples due to variations in the preparing compound, RNA extraction, or reaction performance during labeling or hybridization. Housekeeping transcripts used to analyze miRNAs in tissues (e.g. RNU6 and RNU48) are often unidentifiable in the bloodstream because they are extensively damaged by RNAse mediation.^104,105^ One proposed housekeeping miRNA is miR-16, which has been most frequently cited in previous reports. It is still strongly influenced by hemolysis and cannot be considered a helpful miRNA reference for normalizing data. Several other housekeeping miRNAs have been proposed in independently conducted studies, but there is still a global lack of agreement.^99,106,107^ Universal normalization methods are applied throughout the analysis that determines a reasonably high level of miRNAs. A commonly applied method for amplification-based array data uses a universal estimate of miRNA expression profile, e.g., the mean or median, as a calibrator. However, the diversity of miRNAs found in the bloodstream by PCR-based methods is generally around 100, which may not be sufficient to use a universal normalization method.

 Furthermore, a minimal variety of potential miRNA markers usually need further confirmation after the detection phase, both in independent case series and technically. Nevertheless, post-detection assays (usually performed by assay alone or a custom map with an insufficient number of miRNAs) indeed do not use the same method to normalize the data. To address this issue, Pizzamiglio et al^108^ recently introduced an all-inclusive technique that starts with a data-driven normalization procedure based on amplification-based array data and detects a small number of miRNAs to be used as a reference for normalizing data based on subsequent confirmatory assays. Moreover, Kroh et al^109^ introduced miRNA outright metrology using a standard curve generated by an artificial miRNA (synthetic oligonucleotides) performed by RT-PCR after biological samples. The technique is plausible for quantifying single miRNA markers, but it is not suitable for evaluating complex miRNAs. For microarray-based data, normalization is also a critical step.^110^ miRNA expression is usually profiled using global normalization procedures that have proven successful in gene expression analysis (e.g., Lowness or quantile normalization).

 Nevertheless, the smaller number of functions (compared to the diversity of genes identified in a gene expression microarray) poses some challenges in terms of the relevance of these approaches in the field of miRNA profiling, and newly introduced methods include tailored least-variant set normalization for miRNA microarrays.^111,112^ Quantification of miRNAs based on sequencing is a good novelty compared to qRT-PCR and microarrays, and the optimal procedure for its use is easily standardized. Methods developed for microarray normalization (e.g., lowness or quantile normalization) of miRNA-seq results have also been used.^113^ In addition to the potential procedural biases mentioned above, several other critical variables with a potentially intense impact on the accurate interpretation of circular miRNA biomarker studies are associated with inherent interpersonal variation and the influence of factors independent of disease. When considering circulating miRNAs as biomarker molecules for cancer, the tumor itself is the primary source of variation to be considered. The specificity of miRNA expression patterns for individual cancer types, circulating miRNA signatures are expected to be specific for different cancer types or molecular subtypes and exclusive for tumor functionalities.

## Merits of miRNA in the diagnosis and prognosis of cancers

 Circulating miRNAs can be obtained quickly and without severe injury. Moreover, a plethora of potentially helpful miRNA biomarkers is shown to be stable in normal humans. Although cell-free miRNAs from serum and plasma are among the most common circulating miRNA biomarkers, several other body fluid samples, such as saliva and urine, are also relevant sources of circulating miRNAs.^114^ Overexpression of miR-186-5p has been observed in tumor tissue, urine, and blood of patients with bladder cancer.^115^ Several miRNAs, including miR-210-3p, were upregulated in the urine of patients with transient cell carcinoma and could promote cancer detection.^116^ PCR remains the primary technique for evaluating circulating miRNA. PCR is characterized by amplification as a critical phase that magnifies the main difference between samples, even for relatively insignificant differences. Accordingly, the current detection technique makes circulating miRNAs the most sensitive biomarkers. The miRNAs are formed dynamically and promptly in response to internal or external stimulants, which enhances the ability of miRNAs to be monitored in real-time and dynamically throughout progression changes, from tumor initiation during adhesion to progression.^117,118^

 TNBC patients were found to have considerable downregulation of a miRNA panel consisting of miR-34a-5p, miR-34b-5p and miR-34c-5p. Of the miRNAs mentioned above, the expression of miR-34a-5p and miR-34b-5p were positively correlated with lymph node metastasis and with miR-34c-5p was correlated with tumor grade and distant metastasis.^118^ These findings demonstrate the potential of circulating miRNAs for assessing tumor stage and progression. The dynamically revealed miRNA pattern could illustrate the progression background of cancer throughout its development.

## Conclusion

 MiRNAs are stable molecules in the biological fluids of the body. They have great potential to be considered as non-invasive biomarkers for various cancers because they can be obtained rapidly and with minimal risk in biological body fluids such as saliva, serum, and urine. MiRNAs can be valuable in many ways, including screening for early-stage cancer, subclassification, predicting drug susceptibility, selecting treatment strategies, and screening for tumor chemical or radiological resistance to predict outcomes and recurrence. Significantly, more work needs to be done to fully identify and validate increased or decreased miRNAs in any disease.

## Competing Interests

 The authors declare that there are no conflicts of interest.

## Ethical Approval

 Not applicable.
